# Using Deep Learning to Detect Spinal Cord Diseases on Thoracolumbar Magnetic Resonance Images of Dogs

**DOI:** 10.3389/fvets.2021.721167

**Published:** 2021-11-02

**Authors:** Anika Biercher, Sebastian Meller, Jakob Wendt, Norman Caspari, Johannes Schmidt-Mosig, Steven De Decker, Holger Andreas Volk

**Affiliations:** ^1^Department of Small Animal Medicine and Surgery, University of Veterinary Medicine, Hannover, Germany; ^2^Caspari, Schmidt-Mosig u. Wendt—vetvise GbR, Hannover, Germany; ^3^Department of Clinical Science and Services, Royal Veterinary College, London, United Kingdom

**Keywords:** *Deep Learning*, Convolutional Neural Network (CNN), machine learning, magnetic resonance imaging, spinal cord diseases, myelopathy

## Abstract

*Deep Learning* based *Convolutional Neural Networks* (CNNs) are the state-of-the-art machine learning technique with medical image data. They have the ability to process large amounts of data and learn image features directly from the raw data. Based on their training, these networks are ultimately able to classify unknown data and make predictions. Magnetic resonance imaging (MRI) is the imaging modality of choice for many spinal cord disorders. Proper interpretation requires time and expertise from radiologists, so there is great interest in using artificial intelligence to more quickly interpret and diagnose medical imaging data. In this study, a CNN was trained and tested using thoracolumbar MR images from 500 dogs. T1- and T2-weighted MR images in sagittal and transverse planes were used. The network was trained with unremarkable images as well as with images showing the following spinal cord pathologies: intervertebral disc extrusion (IVDE), intervertebral disc protrusion (IVDP), fibrocartilaginous embolism (FCE)/acute non-compressive nucleus pulposus extrusion (ANNPE), syringomyelia and neoplasia. 2,693 MR images from 375 dogs were used for network training. The network was tested using 7,695 MR images from 125 dogs. The network performed best in detecting IVDPs on sagittal T1-weighted images, with a sensitivity of 100% and specificity of 95.1%. The network also performed very well in detecting IVDEs, especially on sagittal T2-weighted images, with a sensitivity of 90.8% and specificity of 98.98%. The network detected FCEs and ANNPEs with a sensitivity of 62.22% and a specificity of 97.90% on sagittal T2-weighted images and with a sensitivity of 91% and a specificity of 90% on transverse T2-weighted images. In detecting neoplasms and syringomyelia, the CNN did not perform well because of insufficient training data or because the network had problems differentiating different hyperintensities on T2-weighted images and thus made incorrect predictions. This study has shown that it is possible to train a CNN in terms of recognizing and differentiating various spinal cord pathologies on canine MR images. CNNs therefore have great potential to act as a “second eye” for imagers in the future, providing a faster focus on the altered image area and thus increasing workflow in radiology.

## Introduction

Magnetic Resonance Imaging (MRI) has become indispensable in the diagnosis of neurological diseases and is considered the diagnostic tool of choice for spinal cord pathologies of various etiologies. Not only is MRI considered the gold standard for intervertebral disc disease (1), which is among the most common spinal cord disorders in dogs (2), but it also contributes to accurate diagnosis with regard to other spinal cord disorders such as ischemic myelopathies, acute non-compressive nucleus pulposus extrusion (ANNPE), syringomyelia, or spinal cord neoplasia (3–6). The correct interpretation of image data is essential for an accurate diagnosis from which treatment and prognosis are determined. It is common practice in the evaluation of the images to look for certain patterns that could be indicative of a disease.

The principle of *Deep Learning*, a certain type of machine learning, is based on the fact that so-called *Artificial Neural Networks* (ANNs) use large amounts of data to create an algorithm that can ultimately correctly assign unknown data based on its “experience”. In doing so, the network always recognizes the same patterns and thus learns to assign them to certain categories, similar to radiologists. Compared to previous machine learning techniques, *Deep Learning* has the ability to process huge amounts of data and learn image features directly from the raw data. However, *Deep Learning* systems are often referred to as black boxes because it is not possible to identify the criteria used by the network to learn and make predictions (7).

*Convolutional Neural Networks (CNNs)* are the foundation of machine learning with image data. Inspired by the human brain, they process information in a similar way *via* many intermediate elements, also known as neurons. The neurons are part of different layers. The first layer, through which data enters the network, is called *input layer*. This is followed by one or more layers called *hidden layers*. In these layers the data is transformed. Within the layers, all neurons are interconnected. How strongly each neuron is networked with another depends on its *weight*. *Weights* are real numbers and represent adjustable parameters. One could call them control knobs that define the input-output function of the machine. These *weights* are adjusted until the network can make good predictions regarding its training data. Last is the *output layer*, which creates the CNN's prediction. Once the network is successfully trained, it can be applied to unknown data (8–10).

In *supervised learning*, the corresponding data, usually labeled by human experts in the respective field, are provided to the algorithm in a training phase. The annotated data is then considered as the so-called *ground truth* for the algorithm (11). In the case of medical images, networks can be trained to independently make diagnoses or predictions on unknown data and serve as a second-opinion tool for radiologists. The goal is not to dispense with the expertise and experience of radiologists, but to provide a more rapid focus on the altered image area, thus facilitating diagnostic workup. In addition, evidence-based algorithms could also help less experienced imagers make more accurate diagnoses and reduce interobserver variability.

In veterinary medicine, there are already pioneering studies in the imaging field that have taken advantage of the principle of *Deep Learning*. Yoon et al. trained a CNN using canine chest radiographs to discriminate between normal and abnormal findings in terms of cardiac silhouette, lung patterns, position of the mediastinum, and pleural cleft. The CNN was able to make predictions with an accuracy of 92.9–96.9%, sensitivity of 92.1–100%, and specificity of 93.8–96.0% (12).

Using a similar objective and approach, Boissady et al., Burti et al., and Li et al. trained CNNs that successfully detected lesions on canine thoracic radiographs (13–15). In these studies, artificial intelligence (AI) achieved identical or even better diagnostic results compared to radiologists. In the study by Boissady et al., primary thoracic lesions of dogs and cats were detected by the network with a significantly smaller error rate than by radiologists or by radiologists aides by the network (10.7 vs. 16.8 vs. 17.2%).

The study by Burti et al. achieved area under the curve values of >0.9 in the diagnosis of cardiomegaly. In the study by Li et al., the network achieved identical results in terms of accuracy (82.71%), sensitivity (68.42%), and specificity (87.09%) in detecting left atrial enlargement on thoracic radiographs of dogs compared to board certified radiologists.

Banzato et al. developed a CNN to detect degenerative liver disease on ultrasound images of dogs. In addition, the accuracy in diagnosis was to be compared with cytologic liver findings and blood serum biomarkers. The CNN predicted diagnoses with 91% accuracy, 100% sensitivity, and 82.8% specificity, outperforming all other diagnostic tests (16). Further studies by Banzato et al. not only developed a CNN that could successfully distinguish between meningiomas and gliomas on canine cranial MR images (17) but was also able to classify canine meningiomas into different grades (18). A recent study by Spiteri et al. successfully used machine learning to understand neuromorphological changes and identify image-based biomarkers in Cavalier Kings Charles Spaniels with Chiari-like malformation-associated pain and syringomyelia. They concluded that machine learning can aid in the diagnosis of Chiari-like malformation and syringomyelia (19).

The aim of this study was to train a *Deep Learning* based CNN via *supervised learning*, which should be able to detect and discriminate different spinal cord pathologies on thoracolumbar MR images of dogs. It was hypothesized that the CNN would have the ability to detect and discriminate various spinal cord diseases on canine thoracolumbar MR images. This study could provide first insights on the approach, limitations, and sensitivity and specificity of CNNs in MRI interpretation.

## Materials and Methods

### Case Selection

Thoracolumbar MR images of 500 dogs served as the basis for the study in order to generate a sufficiently large data set for the neural network. MR images were obtained from the archives of the Department of Small Animal Medicine and Surgery, University of Veterinary Medicine, Hannover, Germany, between January 2016 and August 2020, as well as from the archives of the Royal Veterinary College, Small Animal Referral Hospital, London, England, between January 2002 and March 2014. Both T1- and T2-weighted images in transverse and sagittal planes, totaling 2,693 images, were used as the basis for network training.

Since the network should be able to distinguish between different spinal cord pathologies, MR images with the following diagnoses were used for network training: unremarkable, intervertebral disc extrusion (IVDE or Hansen Type I), intervertebral disc protrusion (IVDP or Hansen Type II), presumptive fibrocartilaginous embolism (FCE), or acute non-compressive nucleus pulposus extrusion (ANNPE) (were combined due to their similar presentation on MRI; only T2-weighted images were used), syringomyelia and neoplasia. MR images of dogs with disc herniation or protrusion were only included if surgery was performed in order to confirm the suspected pathology and localisation. In the case of neoplasia, a pathologic report (either biopsy or postmortem report) was considered as appropriate evidence. Clinical signs and well-defined MRI variables (3, 4, 20, 21) were considered sufficient evidence for syringomyelia and ANNPE/FCE cases. In this study, the cause of syringomyelia was not further specified. Cases were included in which syringomyelia had spread to the thoracolumbar spinal cord.

### Magnetic Resonance Imaging

MR images were obtained using either a 3.0 T high-field MRI scanner (Achieva 3.0, PhilipsMedical Systems, Best, The Netherlands) or a 1.5 T high-field MRI scanner (Intera 1.5T, Philips Medical Systems, Eindhoven, The Netherlands). All dogs underwent general anesthesia and were positioned in dorsal recumbency during the imaging process.

### Data Set Preparation

All MR images were exported in *Digital Imaging and Communications in Medicine* (DICOM) format and anonymized. In the next step, data from the 500 dogs were divided into a training set (75% or 375 dogs) and a test set (25% or 125 dogs). Randomization of both training and test set was performed *via* www.random.org.

The distribution of the assessed diagnoses within the 500 dogs was as follows: 284 dogs with IVDE (213 for training, 71 for testing), 38 dogs with IVDP (28 for training, 10 for testing), 108 dogs with FCE/ANNPE (81 for training, 27 for testing), 13 dogs with syringomyelia (10 for training, 3 for testing), and 18 dogs with neoplasia (14 for training, 4 for testing), including the following pathologies: meningioma (*n* = 2), hemangiosarcoma (*n* = 2), hemangioma (*n* = 2), multiple myeloma (*n* = 1), nephroblastoma (*n* = 1), osteosarcoma (*n* = 2), lymphoma (*n* = 3), fibrosarcoma (*n* = 1), round cell tumor (*n* = 1), metastases from mammary carcinoma (*n* = 2), and metastases from prostate carcinoma (*n* = 1). Furthermore, 39 dogs with unremarkable MR images (29 for training, 10 for testing) were included. Supplementary Table 1 includes signalment and diagnoses of all 500 dogs.

### Deep Learning Model and Method

The CNN model was trained from scratch using a single-hold-out set and was trained to simultaneously classify multiple output classes. A batch size of 64 was used for training for a total of 15,000 iterations. For training, images were resized to 640 x 640 pixels. For each DICOM image the pixel data of the first accessible frame was exported and converted to a JPEG-Image. Specifically, a YCbCr color space was used together with the provided LUT transformations.

**Figure 1:**
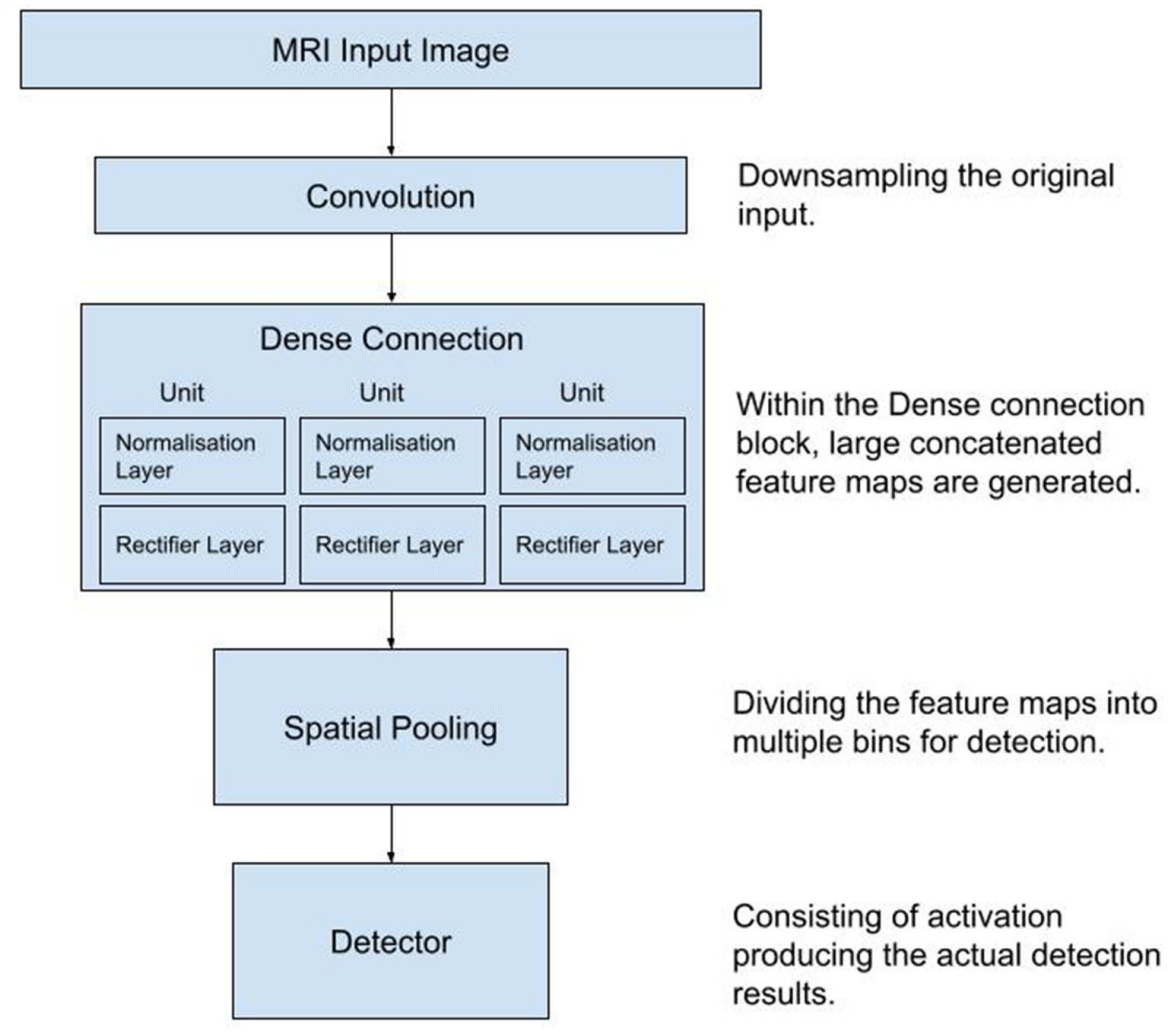
Diagram of the workflow used in the process of data collection, training and testing of the Convolutional Neural Network. MRI, Magnetic Resonance Images.

The architecture of the CNN is shown schematically in Figure 1.

### Network Training and Validation

A platform, which served to upload and mark the data, was programmed exclusively for this project. The anonymized DICOMs from the training set were uploaded into this database and labeled. The labels “extrusion,” “protrusion,” “FCE/ANNPE,” “neoplasia,” and “syringomyelia” were implemented as selection options. The label “edema” was added later to mark hyperintense areas on the MR images that did not show disc herniation or FCEs/ANNPEs. During labeling, the visible pathological alteration was marked by hand if applicable and given the appropriate label. The correct assignment of labels was matched with clinical and pathological reports and observations. This process is called *supervised learning*. If there were several changes on one image, several labels could also be assigned.

After completing the labeling phase of the data, the CNN was first validated and then trained. In this process, the network was trained for each diagnosis individually. During the validation phase, 100 individual images from the training data were randomly selected. In the first step, it was tested whether the CNN was able to distinguish between “lesion” or “no lesion.” This step was repeated and weights were adjusted until satisfactory results were obtained. Next, it was validated whether the CNN can distinguish between different pathologies on the altered images.

The network was trained with a total number of 2,693 images, of which 1,575 images were assigned 1,622 labels (multiple labels could be assigned on one image).

The label edema was assigned 59 times, the label IVDE 833 times, the label IVDP 149 times, the label FCE/ANNPE 379 times, the label neoplasia 167 times and the label syringomyelia 35 times. 1,118 images had no label, thus were unremarkable.

After the training phase was completed, the data from the remaining 125 dogs were used to test how accurate, specific, and sensitive the CNN was in terms of detecting and distinguishing between different spinal cord pathologies.

**Figure 2:**
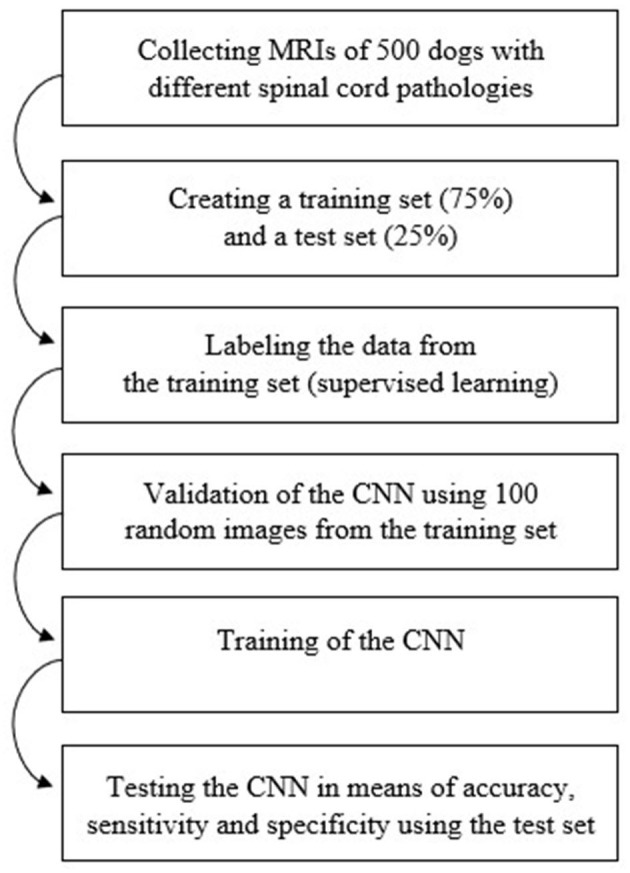
Diagram of the workflow used in order to train and test the Convolutional Neural Network. CNN, Convolutional Neural Network; MRI, magnetic resonance images.

Figure 2 shows the workflow used in the process of data collection, training and testing of the CNN.

## Statistics

Sensitivities, specificities, positive predictive values, negative predictive values, and accuracies were calculated based on contingency tables with true positive, true negative, false positive and false negative decisions per image for each diagnosis, plane, and sequence. 95% confidence intervals (95% CI) for sensitivity, specificity, and accuracy were calculated using the Clopper-Pearson method. 95% CIs for the positive and negative predictive values are the standard logit CIs given by Mercaldo et al. (22). Certainty per image was calculated by the CNN using its training approach. For analysis, the means of the highest certainties given per patient's image set for each correct diagnose, plane and sequence were calculated, if appropriate. In addition, 95% CIs of the mean of certainty were calculated. For the comparison of certainties across planes and sequences per diagnose, one-way ANOVA followed by Holm-Šidák's multiple comparisons test or paired *t*-test were used, where appropriate.

For statistical and graphical analysis, MedCalc (www.medcalc.org) and Prism 9 software from GraphPad (La Jolla, CA, USA) were used. Two-sided tests were used and a *p* ≤ 0.05 was considered significant.

## Results

**Figure 3:**
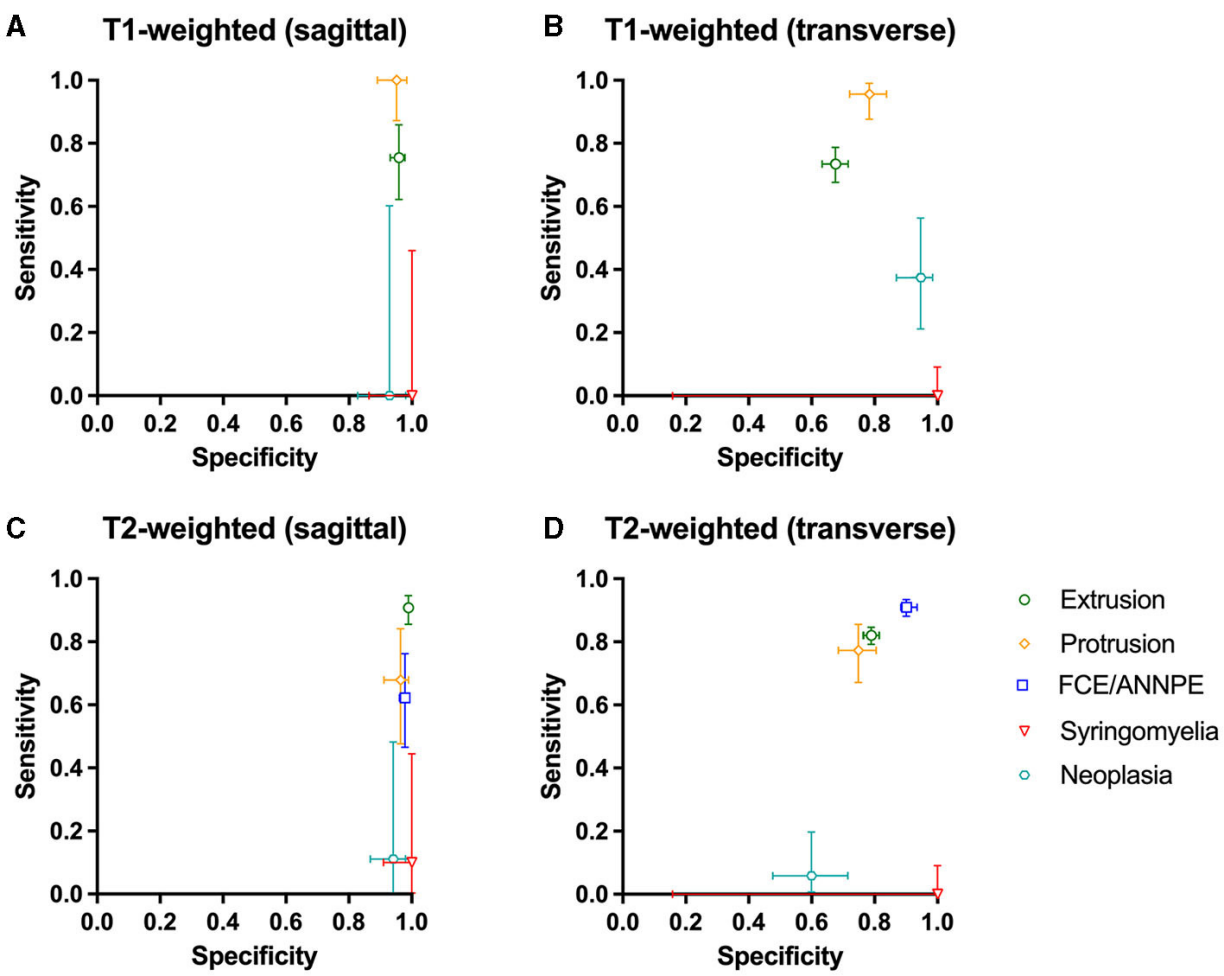
**(A–D)** Sensitivity and specificity of the Convolutional Neural Network for the detection of intervertebral disc extrusion (green circle), protrusion (yellow rhomb), fibrocartilaginous embolism/acute non-compressive nucleus pulposus extrusion (FCE/ANNPE; blue square), syringomyelia (red triangle), and neoplasia (cyan hexagon) on T1-weighted and T2-weighted sagittal and transverse magnetic resonance images of spinal cords of dogs. 95% confidence intervals for sensitivity and specificity are shown with vertical and horizontal bars, respectively.

The network was tested using 7,695 images of 125 dogs. The results can be found in Supplementary Table 2. The network detected IVDEs on sagittal T1-weighted images with a sensitivity of 75.44% [95% CI: 62.24–85.87%] and a specificity of 95.85% [95% CI: 93–97.77%]. On transverse T1-weighted images, sensitivity was 73.46% [95% CI: 67.65–78.73%] and specificity was 67.61% [95% CI: 63.30–71.71%] (Figure 3).

On sagittal T2-weighted images, sensitivity was 90.80% [95% CI: 85.5–94.65%] and specificity was 98.98% [95% CI: 98.18–99.49%], and on transverse T2-weighted images, sensitivity was 82.03% [95% CI: 79.22–84.60%] and specificity was 78.96% [95% CI: 76.56–81.22%] (Figure 3).

The network yielded the following results in the detection of IVDPs: sensitivity of 100% [95% CI: 87.23–100%] on sagittal T1-weighted images and specificity of 95.10% [95% CI: 88.93–98.39%] (Figure 3). On transverse T1-weighted images, it achieved a sensitivity of 95.59% [95% CI: 87.64–99.08%] and a specificity of 78.39% [95% CI: 72.02–83.90%] (Figure 3). On sagittal T2-weighted images, the network achieved a sensitivity of 67.86% [95% CI: 47.65–84.12%] in detecting IVDPs and a specificity of 96.43% [95% CI: 91.11–99.02%] (Figure 3). The sensitivity on transverse T2-weighted images was 77.27% [95% CI: 67.11–85.53%] and the specificity was 74.88% [95% CI: 68.47–80.58%] (Figure 3).

FCE/ANNPE were detected on sagittal T2-weighted images with a sensitivity of 62.22% [95% CI: 46.54–76.23%] and specificity of 97.90% [95% CI: 96.05–99.03%] (Figure 3). Sensitivity on transverse T2-weighted images was 90.98% [95% CI: 88.12–93.35%] and specificity 90.12% [95% CI: 85.66–93.57%] (Figure 3). Sensitivity for detecting syringomyelia on sagittal T1-weighted images was 0% [95% CI: 0.00–45.93%] and specificity was 100% [95% CI: 86.28–100.00%] (Figure 3). Sensitivity on transverse T1-weighted images was 0% [95% CI: 0.00–9.03%] and specificity 100% [95% CI: 15.81–100.00%] (Figure 3).

On sagittal T2-weighted images, the network detected syringomyelia with a sensitivity of 10% [95% CI: 0.25–44.50%] and a specificity of 100% [95% CI: 90.97–100.00%] (Figure 3). The sensitivity on transverse T2-weighted images was 0% [95% CI: 0.00–9.03%] and specificity 100% [95% CI: 15.81–100.00%] (Figure 3). Neoplasia was detected by the network on sagittal T1-weighted images with a sensitivity of 0% [95% CI: 0.00–60.24%] and a specificity of 92.86% [95% CI: 82.71–98.02%] (Figure 3). On transverse T1-weighted images, sensitivity was 37.50% [95% CI: 21.10–56.31%] and specificity was 94.67% [95% CI: 86.90–98.53%] (Figure 3). On sagittal T2-weighted images, sensitivity was 11.11% [95% CI: 0.28–48.25%] and specificity 94.12% [95% CI: 86.90–98.53%] (Figure 3). On transverse T2-weighted images, sensitivity was 5.88% [95% CI: 0.72–19.68%] and specificity was 60.00% [95% CI: 47.59–71.53%] (Figure 3).

Unremarkable images were detected by the network with a specificity of 97.86% on sagittal T1-weighted images and with a specificity of 77.38% on transverse T1-weighted images. On sagittal T2-weighted images, sensitivity was 99% and on transverse T2-weighted images, sensitivity was 81.64% (Supplementary Table 2).

**Figure 4:**
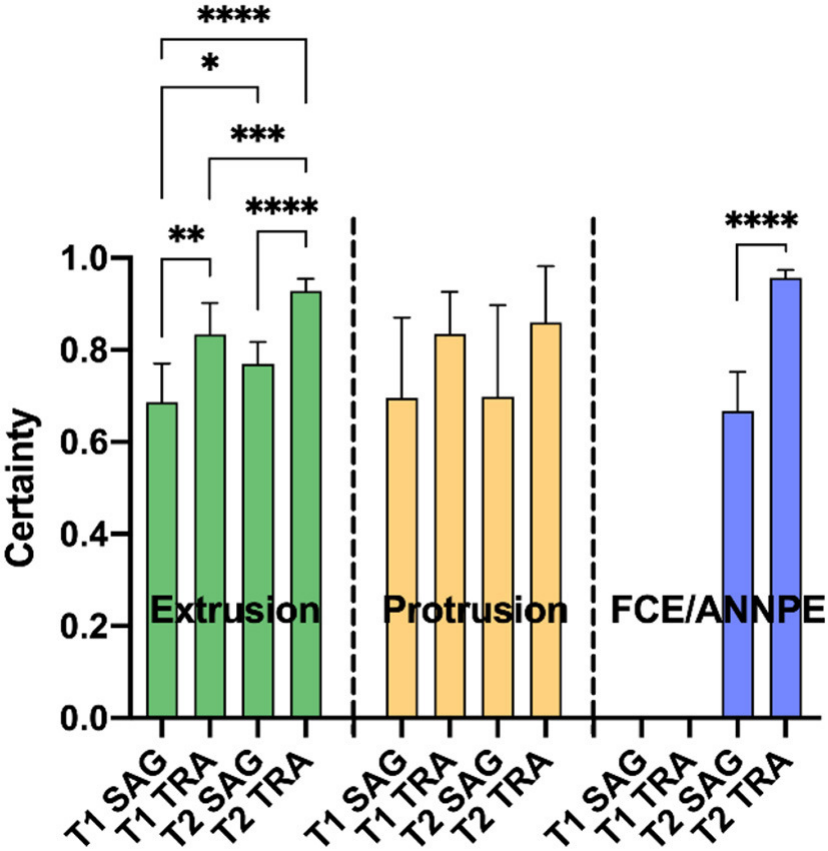
Means of the highest certainties per correct diagnosis [extrusion, protrusion, and fibrocartilaginous embolism/acute non-compressive nucleus pulposus extrusion (FCE/ANNPE)], plane [sagittal (SAG) and transverse (TRA)], and sequence (T1- and T2-weighted) of the Convolutional Neural Network are shown. Bars represent 95% confidence intervals of the mean. In case of extrusion, the detection certainty was significantly higher for transverse MR images compared to sagittal images in each sequence (**p ≤ 0.01 and ****p ≤ 0.0001 for T1- and T2-weighted images, respectively). This also applies for FCE/ANNPE (****p ≤ 0.0001). Extrusions were detected with a significantly higher certainty in T2-weighted images compared to T1-weighted images (*p ≤ 0.05 for sagittal and ***p ≤ 0.01 for transverse images). Concerning extrusions, the system had the highest certainty for transverse T2-weighted and the lowest certainty for sagittal T1-weighted images (****p ≤ 0.0001). No significant differences between planes and sequences were detected for the certainty of protrusions.

In case the network suspected a change on an image, it specified a certainty [0–100%] with which it assumed the respective diagnose on the image (Figure 4). For each animal, the highest certainty was noted and mean values were calculated from the certainties per diagnosis, plane and sequence (Figure 4). Since the network did not perform well in detecting syringomyelias and neoplasias, no mean certainty values were calculated for these diagnoses.

Of all 7,695 images used to test the network, 1,107 images were mislabeled by the CNN, resulting in an error rate of 14.39%. The label IVDE was incorrectly assigned 181 times (16.35%), the label IVDP 27 times (2.44%), the label FCE/ANNPE 397 times (35.86%), the label syringomyelia 5 times (0.45%), the label neoplasia 200 times (18.07%), and the label edema 22 times (1.99%). 276 times (24.93%) an image was incorrectly labeled as inconspicuous.

## Discussion

In this study, a supervised *Deep Learning* based CNN was trained using thoracolumbar transverse or sagittal T1- and T2-weighted MR images from dogs with various myelopathies, including IVDE, IVDP, FCE/ANNPE, syringomyelia and spinal cord neoplasms. Included cases of IVDE, IVDP and neoplasms were either histopathologically or surgically confirmed. After training, the CNN was able to identify IVDE, IVDP and FCE/ANNPE lesions with a high sensitivity and specificity but lacked to reliably detect syringomyelia or neoplastic lesions. Currently, *Deep Learning* with CNNs is considered the state-of-the-art method regarding pattern recognition and classification of image data and has the ability to process huge amounts of data with ease (7), as was the case in our study. Especially since the introduction of cross-sectional imaging, an ever increasing and already vast amount of data is produced every day with a simultaneous lack of time and skilled personnel. Therefore, the desire for automated computer-aided image analysis is apparent. Apart from the opportunities such new tools could bring, there are also limitations, as shown in the current study.

The training and testing data consisted of T1- and T2-weighted sagittal and transverse MR images with the aforementioned diagnoses. In the current study, we trained the CNN only with cases where we had a high certainty in the diagnosis, histopathologically or surgically confirmed. This resulted in a variable data set for the different diseases on which the CNN was trained and tested. The clinically more common diseases were therefore easier identifiable than the not as common ones. In addition, there was no balanced relationship between sagittal and transverse as well as T1- and T2-weighted images because the same number of images is usually not generated for each animal and diagnosis for the corresponding planes and sequences.

The most common diagnosis represented within the entire data set was IVDE, consistent with the fact that disc herniation is the most common spinal cord disease in dogs (2). A total of 284 out of the 500 included dogs were considered to have this diagnosis. As a result, the network has had solid training success. For the sagittal T2-weighted sequences, the network performed very well in detecting disc extrusions with a sensitivity of 90.80% and a specificity of 98.98%. CNN learning is difficult to comprehend as a human, as it is not clear which information is used in the image to differentiate the various pathologies. IVDE often presents as hypointense material in the vertebral canal on T2-weighted images (1, 23), which could help to distinguish them from pathologies that induce hyperintensity on T2-weighted images like FCEs/ANNPEs or syringomyelia. Saying this, this would only be the case on single images in the same series, as on other images spinal cord edema secondary to IVDE could be depicted as hyperintense lesion on T2-weighted images. Hyperintensities on T2-weighted images are frequently recognized for various pathologies. This fact in combination with a small training data set could have resulted in the poor performance for detecting syringomyelia and neoplastic lesions. The more data a network is trained on, the better the detection rate. A potential further limitation of this study, which should be considered was that the MR images used as the basis of this study were generated from two different MRI machines with different field strengths. To what extent and if this fact had an impact on the performance of the trained network is unknown. Future studies need to explore how this can influence training and also the application of CNNs in practice.

IVDP can be differentiated from IVDE and is relatively easy diagnosed on MR images (24). IVDPs are often more midline instead of lateralized and have a partial disc degeneration or partial nucleus pulposus dehydration. On the other hand, IVDE affects usually only one disc space and can have dispersed material over multiple spaces. The CNN needed not as many images as for IVDE to be trained and detect reliably IVDP on the test series. It can be assumed that as clearer the pathology presents on MR images, the less amount of image data is needed to train the network. IVDPs present more consistently on MRI than IVDEs because IVDEs may present right-sided, left-sided, dorsal, or ventral. In addition, the prolapsed disc material may spread across multiple intervertebral spaces and be of mixed signal intensity.

A study by De Decker et al. evaluated MRI guidelines for differentiation between thoracolumbar IVDE and IVDP in dogs. Diagnostic accuracy was 79.6% after application of the guidelines and interobserver agreement was moderate (kappa = 0.41). In addition, diagnostic accuracy was significantly dependent on the experience of the observer (25). In this study, the CNN achieved good to very good results with regard to the accuracy with which the diagnoses IVDE and IVDP were made. *Deep Learning* based second opinion tools could therefore contribute to significantly improve diagnoses in the future and reduce intra- and inter-observer variability.

In addition, the success of the network is largely dependent on the supervisor, who labels the data and thus provides the basis for the algorithm (7). Nevertheless, the training data are usually verified by clinical, surgical, and pathological reports from different experts, which supports the decision of the labeler and thus provides a high degree of certainty for the adequate “training method.”

This study shows that training success can be achieved even with a small amount of data, as long as the individual labels are not too similar. If certain patterns within the data are too similar, training success cannot be achieved with a small amount of data, as can be seen with the diagnoses “neoplasia” and “syringomyelia.” In addition to the small amount of training data for neoplasia, their expression can be very different among patients concerning their morphology and their tissue of origin (5). This makes it extremely difficult for the algorithm to discern unique image characteristics of this diagnosis. In this study, all types of spinal cord tumors were grouped together and not differentiated. It might be possible to train a CNN with significantly more MR images of spinal cord tumors, not only in terms of detecting but also distinguishing the tumor type. This might provide a challenge in veterinary medicine as histopathologically confirmed data sets would be difficult to retrieve and non-confirmed data set would be suboptimal.

In this study, adding spinal cord tumors did not add value to the overall success of the CNN. Among all mislabeled images, the label “neoplasia” was the second most common mislabeled image, accounting for 18.07%. In order to train the CNN with images of spinal cord neoplasms, post-contrast images were intentionally omitted to evaluate whether the algorithm could detect altered structures without contrast agent. However, as a result, the CNN incorrectly assigned the label “neoplasia” more frequently. Overall, the network showed little success in detecting spinal cord tumors on any sequences.

Regarding the diagnosis of syringomyelia, the CNN obtained the worst results. Although syringomyelia is fundamentally very different from the other pathologies due to its relatively wide, fluid filled cavities in the spinal cord, the CNN incorrectly defined them most often as “inconspicuous.” This can likely be explained by the fact that syringomyelia resemble the epidural fat in parasagittal cross-sectional T2-weighted images, which also appears to be hyperintense (26). In addition, the network was trained with only 35 images labeled “syringomyelia.” This is due to the fact that only thoracolumbar MR images were used for training in this study, and syringomyelia do not in all cases extend into the thoracolumbar spinal cord. On transverse MR images, syringomyelia were often confused with FCEs/ANNPEs because the network was trained with significantly more images of FCEs and therefore may have recognized the central hyperintensity in the spinal cord as latter more frequently. With a larger data set of syringomyelia, it might be probable that the CNN could better distinguish different hyperintensities.

With regard to the detection of FCEs and ANNPEs, which were combined in this study, the CNN performed very well, especially on transverse T2-weighted images. FCEs and ANNPEs accounted for the second largest proportion of training data, which is reflected in the training success. At the same time, the FCE/ANNPE label was the most common misattributed label, accounting for 35.86% of all misattributed labels. Other causes of hyperintensity in the spinal cord than FCE/ANNPE would include syringomyelia or edema due to disc herniation (27). However, the separate label “edema” was introduced for this event as well. In principle, the CNN is very efficient at detecting hyperintensities in the spinal cord but cannot necessarily always differentiate them.

In this study, FCEs and ANNPEs were combined and not differentiated. However, Fenn et al. showed that inter-observer agreement to differentiate FCEs and ANNPEs on MR images based on certain criteria was moderate (kappa = 0.56) and intra-observer agreement was moderate to good (kappa = 0.47 and 0.79, respectively) (28). This demonstrates that even board-certified veterinary radiologists and neurologists may have difficulty assigning hyperintensities on MR images to the correct pathologies. In contrast, another study by Specchi et al. yielded excellent inter-observer agreement with respect to the differentiation of FCEs and ANNPEs on MRI using directional patterns and length of intramedullary hyperintensity on T2-weighted MR images as well as enhancement patterns in postcontrast T1-weighted MR images as criteria (21).

In future, CNNs could relate image data to signalment and clinical history simultaneously and better results in differentiating various pathologies could be obtained. For example, ANNPEs are often associated with traumatic events and FCEs with a peracute onset of non-progressive and non-painful clinical signs (3, 28), whereas syringomyelia is particularly common in certain breeds and develops because of congenital anomalies, leading to spinal pain (6). It is often necessary to add other sequences in addition to T1- and T2-weighted images, for example, to differentiate hemorrhage or tissue-bound from free fluid. Since this network was trained using only T1- and T2-weighted images, it would reach its limits. However, in principle, it would be possible to train a network using further sequences, which could be evaluated in future studies.

Several studies on spinal cord diseases have shown that there are not only discrepancies in the interpretation of MR images between radiologists with different levels of experience, but also that the same imager can make different diagnoses at different time points on the same MR images (25, 28). Well-trained evidence-based algorithms could contribute to less inter- and intra-observer variability in practical application and contribute to a more accurate diagnosis as a computer-assisted second opinion. So far, this network is only able to mark the assumed altered area and make a prediction regarding its pathology. Closer image analysis with respect to localization in the vertebral column would have to be performed by human expertise. Particularly in the case of disc herniations worthy of surgery, it is important in practice to analyze the MR images with respect to the side of the disc herniation and to determine over how many intervertebral spaces the prolapsed disc material extends. The presence of blood also influences surgical planning (29).

Another advantage of the CNN, however, is that it assigns a certainty to its predictions. Similarly, a radiologist would make certain diagnoses with a high degree of certainty or may be uncertain about a diagnosis. If the CNN is used as a second opinion tool, the CNN's diagnosis can be better assessed on the basis of the certainty. Regarding this study, the network made diagnoses on transverse MR images with higher certainties than on sagittal MR images.

Overall, the training success of the CNN regarding the results of sensitivities, specificities, certainties and an error rate of 14.39% can be considered successful and suggests that this CNN is capable of detecting and differentiating various spinal cord pathologies. Since this CNN was trained with a total of 1,622 labels, extended training with more labels could lead to an even better performance of the network.

Limitations of this study are the comparatively small data set with an uneven distribution of each pathological entity, resulting in different sensitivities and specificities for detecting each pathology. Future studies could evaluate whether CNNs would achieve better results in detecting and differentiating various spinal cord pathologies with larger data sets.

## Conclusion

*Deep Learning*-based CNNs have the potential to successfully learn and make predictions based on MR images in terms of detecting and distinguishing between different spinal cord pathologies in dogs. In this study, despite a limited data set, the diagnoses IVDE, IVDP, and FCE/ANNPE could be detected and distinguished from each other. In contrast, the success of the predictions regarding the diagnoses “syringomyelia” and “neoplasia” was insufficient. Since the success of the network is largely dependent on the number of training data, larger data sets should be used in future studies. Future studies also need to explore the influence of the field strength and the brand of the MRI machine on the performance of CNNs.

CNNs should not be viewed as the sole diagnostic tool, but rather as a “second eye.” In this context, CNNs can be trained with almost infinite data and their learning potential can therefore be increased immeasurably over time.

## Data Availability Statement

The raw data supporting the conclusions of this article will be made available by the authors, without undue reservation.

## Ethics Statement

The study was conducted in accordance with the German Animal Welfare Act within the law of animal welfare, following the ethical guidelines of the University of Veterinary Medicine, Hannover (approval by the thesis commission and the data protection officer). Written informed consent was obtained from the owners for the participation of their animals in this study.

## Author Contributions

AB compiled the clinical data, labeled the imaging data set, and was actively involved in drafting the manuscript. HV compiled the manuscript and trained AB to correctly label the images. AB, SM, and HV designed the study, interpreted the statistical analysis, and were involved in data presentation. JW, NC, and JS-M were involved in providing access to and training of CNN. SD provided and interpreted clinical data from the Royal Veterinary College. All authors contributed to revisions of manuscript and approved the submitted version.

## Funding

This manuscript was financially supported by the University of Veterinary Medicine, Hannover, through Open Access Publishing funding.

## Conflict of Interest

JW, NC, and JS-M own vetvise GbR and were involved in training the CNN. The commercial partner has not been involved in case recruitment and statistical analysis of the results. Furthermore, the commercial partner could not prevent the manuscript from being submitted for publication. The remaining authors declare that the research was conducted in the absence of any commercial or financial relationships that could be construed as a potential conflict of interest.

## Publisher's Note

All claims expressed in this article are solely those of the authors and do not necessarily represent those of their affiliated organizations, or those of the publisher, the editors and the reviewers. Any product that may be evaluated in this article, or claim that may be made by its manufacturer, is not guaranteed or endorsed by the publisher.
