# Supplementary material for: Using Deep Learning to Detect Spinal Cord Diseases on Thoracolumbar Magnetic Resonance Images of Dogs
Source: Front Vet Sci. 2021 Nov 2;8:721167. doi: 10.3389/fvets.2021.721167 (PMC8593183; doi:10.3389/fvets.2021.721167)
Supplement: Supplementary file 1 [file Table_1.docx]

**Supplementary Table 1: Breed, age (in months), sex, weight, definition, and localisation of spinal cord lesion of the 500 dogs whose magnetic resonance images were used to train and test the network.**

*FCE =* fibrocartilaginous embolism*; ANNPE =* acute non-compressive nucleus pulposus extrusion*; Th =* thoracic spinal cord segment*;*

*L =* lumbar spinal cord segment*.*

*Blank fields: no data*

| **dog** | **breed** | **age (month)** | **gender (0=male**  **1=female)** | **neutered (0=intact,**  **1= neutered)** | **weight** | **lesion localisation** | **comment** | **type of disease (1=extrusion,**  **2=protrusion,**  **3= neoplasia,**  **4=syringomyelia,**  **5= FCE/ANNPE)** |
| --- | --- | --- | --- | --- | --- | --- | --- | --- |
| **1** | Basset | 85 | 0 | 0 | 29,4 kg | Th11-12 |  | 1 |
| **2** | Beagle | 130 | 0 | 1 | 19,5 kg | Th12/13, L1/2 |  | 1 |
| **3** | Beagle | 48 | 1 | 1 | 8,3 kg | L3/4 |  | 1 |
| **4** | Beagle | 133 | 1 | 1 | 16,9 kg | Th12/13 |  | 1 |
| **5** | Beagle | 92 | 1 | 1 | 20,4 kg | Th11-13 |  | 1 |
| **6** | Beagle | 129 | 0 | 1 | 16,7 kg | Th13/L1 |  | 1 |
| **7** | Cavalier Kings Charles Spaniel | 105 | 1 | 1 | 9,0 kg | Th11-13 |  | 1 |
| **8** | Cocker Spaniel | 144 | 0 | 0 | 11,5 kg | Th13/L1 |  | 1 |
| **9** | Cocker Spaniel | 74 | 0 | 0 | 13,6 kg | Th12/13 |  | 1 |
| **10** | Cocker Spaniel | 144 | 0 | 0 | 13,6 kg | Th13/L1 |  | 1 |
| **11** | Cocker Spaniel | 84 | 1 | 1 |  | L2/L3 |  | 1 |
| **12** | Cocker Spaniel | 95 | 0 | 1 | 11,0 kg | Th12/13 |  | 1 |
| **13** | Cocker Spaniel | 53 | 0 | 1 |  | Th12/13 |  | 1 |
| **14** | Cocker Spaniel | 56 | 1 | 1 | 12,3 kg | L6/L7 |  | 1 |
| **15** | Cocker Spaniel | 102 | 1 | 1 |  | L3/L4 |  | 1 |
| **16** | Cocker Spaniel | 96 | 0 | 1 | 14,1 kg | L7/S1 |  | 1 |
| **17** | Cocker Spaniel | 46 | 0 | 1 | 11,25 kg | Th12/13 |  | 1 |
| **18** | Cocker Spaniel | 140 | 0 | 1 |  | Th13/L1 |  | 1 |
| **19** | Cocker Spaniel | 86 | 1 | 1 | 9,9 kg | Th13/L1 |  | 1 |
| **20** | Cocker Spaniel | 43 | 1 | 1 |  | L3/L4 |  | 1 |
| **21** | Cocker Spaniel | 169 | 1 | 0 | 16,7 kg | Th13/L1 |  | 1 |
| **22** | Cocker Spaniel | 130 | 1 | 1 | 14,0 kg | L2/L3 |  | 1 |
| **23** | Cocker Spaniel | 68 | 0 | 1 |  | Th12/13 |  | 1 |
| **24** | Cocker Spaniel | 120 | 0 | 1 |  | Th13/L1 |  | 1 |
| **25** | Cocker Spaniel | 47 | 1 | 1 |  | L5/L6 |  | 1 |
| **26** | Cocker Spaniel | 65 | 1 | 1 | 12,4 kg | L3/L4 |  | 1 |
| **27** | Cocker Spaniel | 126 | 0 | 1 | 13,6 kg | L2/L3 |  | 1 |
| **28** | Cocker Spaniel | 66 | 1 | 1 | 10,1 kg | Th12/13 |  | 1 |
| **29** | Cocker Spaniel | 48 | 0 | 1 | 20,0 kg | L5/L6 |  | 1 |
| **30** | Cocker Spaniel | 120 | 1 | 1 | 20,0 kg | Th12/13 |  | 1 |
| **31** | Cocker Spaniel | 59 | 1 | 1 |  | L3/L4 |  | 1 |
| **32** | Cocker Spaniel | 70 | 0 | 1 | 14,6 kg | Th13/L1 |  | 1 |
| **33** | Cocker Spaniel | 66 | 0 | 0 | 13,1 kg | L4/5 |  | 1 |
| **34** | Cocker Spaniel | 49 | 0 | 1 | 18,5 kg | L2/L3 |  | 1 |
| **35** | Cocker Spaniel | 59 | 1 | 1 |  | L3/L4 |  | 1 |
| **36** | Cocker Spaniel | 86 | 1 | 1 | 12,5 kg | L6/7 |  | 1 |
| **37** | Cocker Spaniel | 65 | 1 | 1 |  | Th12/13 |  | 1 |
| **38** | Cocker Spaniel | 40 | 0 | 0 | 13,0 kg | L5/L6 |  | 1 |
| **39** | Cocker Spaniel | 60 | 0 | 0 |  | Th11/Th12 |  | 1 |
| **40** | Cocker Spaniel | 103 | 0 | 1 | 15,0 kg | L5/L6 |  | 1 |
| **41** | Cocker Spaniel | 52 | 1 | 1 | 12,0 kg | L6/L7 |  | 1 |
| **42** | Cocker Spaniel | 115 | 0 | 1 | 14,0 kg | L6/L7 |  | 1 |
| **43** | Cocker Spaniel | 116 | 0 | 1 |  | L4/L5 |  | 1 |
| **44** | Cocker Spaniel | 87 | 0 | 1 | 14,0 kg | L3/L4 |  | 1 |
| **45** | Cocker Spaniel | 48 | 0 | 1 | 13,0 kg | Th12/13 |  | 1 |
| **46** | Cocker Spaniel | 115 | 0 | 1 |  | L2/L3 |  | 1 |
| **47** | Cocker Spaniel | 74 | 0 | 1 |  | L6/L7 |  | 1 |
| **48** | Cocker Spaniel | 106 | 1 | 1 |  | L2/L3 |  | 1 |
| **49** | Cocker Spaniel | 53 | 0 | 1 | 15,0 kg | L3/L4 |  | 1 |
| **50** | Cocker Spaniel | 66 | 0 | 1 | 17,0 kg | Th11/12 |  | 1 |
| **51** | Cocker Spaniel | 54 | 0 | 1 | 12,0 kg | L6/7 |  | 1 |
| **52** | Cocker Spaniel | 135 | 0 | 1 | 18,0 kg | L5-L7 |  | 1 |
| **53** | Cocker Spaniel | 46 | 0 | 1 | 11,4 kg | L2/L3 |  | 1 |
| **54** | Dachshund | 70 | 1 | 0 | 11,6 kg | Th11/12 |  | 1 |
| **55** | Dachshund | 51 | 0 | 1 | 8,6 kg | Th12/13 |  | 1 |
| **56** | Dachshund | 112 | 0 | 0 | 13,8 kg | Th12/13 |  | 1 |
| **57** | Dachshund | 70 | 1 | 1 | 5,2 kg | Th11-Th13 |  | 1 |
| **58** | Dachshund | 50 | 0 | 0 | 6,0 kg | Th11/12 |  | 1 |
| **59** | Dachshund | 90 | 1 | 1 | 10,6 kg | Th12-L2 |  | 1 |
| **60** | Dachshund | 93 | 0 | 1 | 7,9 kg | Th10-Th12 |  | 1 |
| **61** | Dachshund | 135 | 0 | 1 | 10,9 kg | Th12/13 |  | 1 |
| **62** | Dachshund | 24 | 1 | 0 | 4,4 kg | Th11/12 |  | 1 |
| **63** | Dachshund | 95 | 0 | 1 | 9,2 kg | Th12/13 |  | 1 |
| **64** | Dachshund | 60 | 0 | 1 | 10,9 kg | Th12/13 |  | 1 |
| **65** | Dachshund | 162 | 0 | 1 | 9,8 kg | L1-L3 |  | 1 |
| **66** | Dachshund | 81 | 0 | 0 | 5,2 kg | Th10/11 |  | 1 |
| **67** | Dachshund | 47 | 0 | 0 | 9,6 kg | Th9-Th11 |  | 1 |
| **68** | Dachshund | 83 | 0 | 0 | 7,0 kg | Th11/12 |  | 1 |
| **69** | Dachshund | 147 | 0 | 0 | 11,0 kg | L3/4 |  | 1 |
| **70** | Dachshund | 59 | 0 | 0 | 7,1 kg | Th13/L1 |  | 1 |
| **71** | Dachshund | 64 | 0 | 0 | 3,8 kg | Th9-Th12, Th13/L1 |  | 1 |
| **72** | Dachshund | 88 | 0 | 1 | 4,7 kg | Th11/12 |  | 1 |
| **73** | Dachshund | 59 | 1 | 0 | 6,2 kg | L1/L2 |  | 1 |
| **74** | Dachshund | 79 | 0 | 0 | 11,0 kg | L4/L5 |  | 1 |
| **75** | Dachshund | 42 | 1 | 1 | 12,0 kg | Th9-Th12 |  | 1 |
| **76** | Dachshund | 72 | 0 | 1 | 8,1 kg | L3-L5 |  | 1 |
| **77** | Dachshund | 37 | 1 | 1 | 5,2 kg | Th13-L2 |  | 1 |
| **78** | Dachshund | 112 | 0 | 0 | 6,0 kg | L3/L4 |  | 1 |
| **79** | Dachshund | 59 | 1 | 1 | 10,0 kg | L4/5 |  | 1 |
| **80** | Dachshund | 89 | 1 | 0 | 8,9 kg | Th11-13 |  | 1 |
| **81** | Dachshund | 49 | 1 | 1 | 7,6 kg | L1-L3 |  | 1 |
| **82** | Dachshund | 109 | 0 | 1 | 10,2 kg | L1/L2 |  | 1 |
| **83** | Dachshund | 27 | 1 | 1 | 7,3 kg | L3-L5 |  | 1 |
| **84** | Dachshund | 117 | 0 | 0 | 10,4 kg | Th11-13 |  | 1 |
| **85** | Dachshund | 66 | 1 | 0 | 10,5 kg | Th13-L2 |  | 1 |
| **86** | Dachshund | 65 | 0 | 0 | 8,0 kg | Th13/L1 |  | 1 |
| **87** | Dachshund | 61 | 1 | 1 | 8,0 kg | Th11/12 |  | 1 |
| **88** | Dachshund | 103 | 0 | 0 | 8,3 kg | Th13/L1, L2/3 |  | 1 |
| **89** | Dachshund | 123 | 1 | 0 | 6,0 kg | L1/2 (8LW) |  | 1 |
| **90** | Dachshund | 50 | 0 | 0 | - | L1/L2 |  | 1 |
| **91** | Dachshund | 58 | 1 | 1 | 5,2 kg | L3/L4 |  | 1 |
| **92** | Dachshund | 46 | 1 | 1 | 5,8 kg | Th13-L3 |  | 1 |
| **93** | Dachshund | 51 | 1 | 1 | 5,9 kg | Th11/12 |  | 1 |
| **94** | Dachshund | 83 | 1 | 1 | 8,2 kg | Th13/L1 |  | 1 |
| **95** | Dachshund | 54 | 1 | 1 | 9,8 kg | Th12/13 |  | 1 |
| **96** | Dachshund | 50 | 1 | 1 | 5,9 kg | Th12/13 |  | 1 |
| **97** | Dachshund | 93 | 1 | 1 | 5,7 kg | Th11/12 |  | 1 |
| **98** | Dachshund | 72 | 0 | 1 |  | Th12/Th13 |  | 1 |
| **99** | Dachshund | 104 | 0 | 1 |  | Th11/12 |  | 1 |
| **100** | Dachshund | 124 | 1 | 1 | 9,2 kg | L2/L3 |  | 1 |
| **101** | Dachshund | 183 | 0 | 1 |  | Th13/L1 |  | 1 |
| **102** | Dachshund | 22 | 0 | 1 | 5,4 kg | Th13/L1 |  | 1 |
| **103** | Dachshund | 69 | 0 | 0 | 6,4 kg | Th13/L1 |  | 1 |
| **104** | Dachshund | 84 | 1 | 1 |  | L1/L2 |  | 1 |
| **105** | Dachshund | 65 | 1 | 1 | 3,4 kg | Th11/12 |  | 1 |
| **106** | Dachshund | 24 | 0 | 0 | 5,9 kg | Th13/L1 |  | 1 |
| **107** | Dachshund | 47 | 0 | 0 | 5,2 kg | L5/L6 |  | 1 |
| **108** | Dachshund | 93 | 0 | 0 | 7,5 kg | Th11/12 |  | 1 |
| **109** | Dachshund | 41 | 0 | 1 | 6,4 kg | L3/L4 |  | 1 |
| **110** | Dachshund | 39 | 0 | 1 |  | L1/L2 |  | 1 |
| **111** | Dachshund | 59 | 1 | 1 | 6,2 kg | Th13/L1 |  | 1 |
| **112** | Dachshund | 41 | 0 | 1 |  | L1/l2 |  | 1 |
| **113** | Dachshund | 72 | 1 | 1 |  | Th12/13 |  | 1 |
| **114** | Dachshund | 66 | 0 | 1 | 7,0 kg | Th9/10 |  | 1 |
| **115** | Dachshund | 54 | 1 | 1 |  | Th12-L1 |  | 1 |
| **116** | Dachshund | 50 | 1 | 0 | 5,4 kg | Th13/L1 |  | 1 |
| **117** | Dachshund | 54 | 0 | 0 | 4,4 kg | Th11/12 |  | 1 |
| **118** | Dachshund | 72 | 0 | 1 |  | Th13/L1 |  | 1 |
| **119** | Dachshund | 62 | 1 | 1 | 9,4 kg | Th13/L1 |  | 1 |
| **120** | Dachshund | 66 | 0 | 1 | 5,2 kg | Th12/13 |  | 1 |
| **121** | Dachshund | 108 | 0 | 0 | 8,9 kg | L2/L3 |  | 1 |
| **122** | Dachshund | 79 | 1 | 1 | 5,9 kg | Th12/13 |  | 1 |
| **123** | Dachshund | 72 | 1 | 1 | 6,4 kg | Th13/L1 |  | 1 |
| **124** | Dachshund | 38 | 1 | 1 | 5,2 kg | Th12/13 |  | 1 |
| **125** | Dachshund | 66 | 1 | 1 | 7,8 kg | Th13/L1 |  | 1 |
| **126** | Dachshund | 109 | 0 | 1 | 8,6 kg | Th12/13 |  | 1 |
| **127** | Dachshund | 78 | 0 | 1 |  | L2/L3 |  | 1 |
| **128** | Dachshund | 91 | 0 | 1 | 9,7 kg | Th11/12 |  | 1 |
| **129** | Dachshund | 47 | 1 | 1 | 5,4 kg | L4/L5 |  | 1 |
| **130** | Dachshund | 84 | 0 | 1 | 8,2 kg | Th12/13 |  | 1 |
| **131** | Dachshund | 91 | 1 | 1 | 6,0 kg | Th12/13 |  | 1 |
| **132** | Dachshund | 46 | 0 | 0 | 6,5 kg | Th13/L1 |  | 1 |
| **133** | Dachshund | 66 | 0 | 1 | 5,75 kg | Th12/13 |  | 1 |
| **134** | Dachshund | 85 | 0 | 1 |  | L3/L4 |  | 1 |
| **135** | Dachshund | 55 | 1 | 1 | 5,6 kg | Th12-L1 |  | 1 |
| **136** | Dachshund | 35 | 1 | 1 |  | L2/L3 |  | 1 |
| **137** | Dachshund | 96 | 0 | 0 | 8,2 kg | Th13/L1 |  | 1 |
| **138** | Dachshund | 67 | 1 | 0 | 5,0 kg | Th12/13 |  | 1 |
| **139** | Dachshund | 72 | 1 | 1 | 6,7 kg | Th12-L2 |  | 1 |
| **140** | Dachshund | 110 | 0 | 1 | 5,7 kg | L1/L2 |  | 1 |
| **141** | Dachshund | 100 | 0 | 1 | 7,0 kg | L3/L4 |  | 1 |
| **142** | Dachshund | 74 | 0 | 0 | 4,7 kg | Th12/13 |  | 1 |
| **143** | Dachshund | 40 | 1 | 1 | 5,9 kg | L3/L4 |  | 1 |
| **144** | Dachshund | 54 | 1 | 1 | 7,3 kg | Th13/L1 |  | 1 |
| **145** | Dachshund | 106 | 0 | 1 | 9,8 kg | Th13/L1 |  | 1 |
| **146** | Dalmatian | 83 | 0 | 0 | 31,9 kg | Th11-L4 |  | 1 |
| **147** | Dalmatian | 138 | 0 | 0 | 24,4 kg | L1-L3 |  | 1 |
| **148** | French Bulldog | 77 | 0 | 1 | 12,4 kg | L2-L4 |  | 1 |
| **149** | French Bulldog | 62 | 1 | 1 | 10,0 kg | L4/L5 |  | 1 |
| **150** | French Bulldog | 55 | 0 | 0 | 15,7 kg | L1-L4 |  | 1 |
| **151** | French Bulldog | 36 | 0 | 0 | 9,9 kg | L1/2 |  | 1 |
| **152** | French Bulldog | 42 | 0 | 0 | 15,0 kg | Th11-L3 |  | 1 |
| **153** | French Bulldog | 29 | 0 | 0 | 14,0 kg | L1-L3 |  | 1 |
| **154** | French Bulldog | 67 | 1 | 1 | 15,8 kg | L2/L3 |  | 1 |
| **155** | French Bulldog | 32 | 0 | 0 | 13,5 kg | L1-L4 |  | 1 |
| **156** | French Bulldog | 41 | 0 | 1 | 12,9 kg | L2-L3 |  | 1 |
| **157** | French Bulldog | 28 | 0 | 1 | 15,5 g | L1/L2 |  | 1 |
| **158** | French Bulldog | 75 | 1 | 1 | 12,3 kg | L3/L4 |  | 1 |
| **159** | French Bulldog | 42 | 0 | 0 | 13,8 kg | L2/L3 |  | 1 |
| **160** | French Bulldog | 45 | 0 | 0 | 12,1 kg | L3-L6 |  | 1 |
| **161** | French Bulldog | 29 | 0 | 0 | 13,8 kg | L2-L5 |  | 1 |
| **162** | French Bulldog | 46 | 1 | 0 | 10,0 kg | Th12-L2 |  | 1 |
| **163** | French Bulldog | 26 | 0 | 1 | 6,6 kg | Th11-Th13 |  | 1 |
| **164** | French Bulldog | 30 | 1 | 1 | 10,0 kg | Th10-Th13 |  | 1 |
| **165** | French Bulldog | 42 | 0 | 0 | 16,3 kg | Th13-L3 |  | 1 |
| **166** | French Bulldog | 47 | 0 | 0 | 17,5 kg | L1/L2 |  | 1 |
| **167** | French Bulldog | 19 | 0 | 1 | 15,7 kg | Th12-L1, L3/4 |  | 1 |
| **168** | French Bulldog | 68 | 1 | 1 | 7,9 kg | L4/L5 |  | 1 |
| **169** | French Bulldog | 77 | 0 | 0 | 14,4 kg | L4-L6 |  | 1 |
| **170** | French Bulldog | 32 | 0 | 0 | 14,2 kg | L2/3 |  | 1 |
| **171** | French Bulldog | 81 | 1 | 1 | 13,0 kg | L1/L2 |  | 1 |
| **172** | French Bulldog | 40 | 1 | 1 | 12,7 kg | Th13-L4 |  | 1 |
| **173** | French Bulldog | 34 | 0 | 1 | 13,6 kg | L4-L6 |  | 1 |
| **174** | German Shepherd | 130 | 0 | 1 | 35,3 kg | Th2-Th4 |  | 1 |
| **175** | German Shepherd | 55 | 0 | 1 | 31,0 kg | Th13/L1 |  | 1 |
| **176** | Hanover hound | 35 | 0 | 0 | 33,0 kg | L3/L4 |  | 1 |
| **177** | Havanese | 53 | 1 | 1 | 7,1 kg | Th12-L1 |  | 1 |
| **178** | Havanese | 106 | 0 | 1 | 5,6 kg | Th13-L2 |  | 1 |
| **179** | Havanese | 74 | 1 | 0 | 5,7 kg | Th12/13 |  | 1 |
| **180** | Jack Russell Terrier | 109 | 0 | 0 | 11,3 kg | L3/L4 |  | 1 |
| **181** | Jack Russell Terrier | 57 | 0 | 0 | 6,5 kg | Th12-L1 |  | 1 |
| **182** | Jack Russell Terrier | 47 | 0 | 1 | 10,4 kg | L2/L3 |  | 1 |
| **183** | Jack Russell Terrier | 108 | 0 | 1 | 8,6 kg | Th11/12 |  | 1 |
| **184** | Jack Russell Terrier | 55 | 1 | 1 | 6,0 kg | Th12-L1 |  | 1 |
| **185** | Jack Russell Terrier | 59 | 1 | 1 | 7,4 kg | Th11/12 |  | 1 |
| **186** | Kopov Bracke | 58 | 1 | 1 | 20,0 kg | Th11-L1 |  | 1 |
| **187** | Labrador | 103 | 1 | 0 | 21,5 kg | L4/L5 |  | 1 |
| **188** | Labrador | 38 | 1 | 0 | 33,5 kg | Th12/13 |  | 1 |
| **189** | Labrador | 134 | 0 | 0 | 42,2 kg | Th13-L4 |  | 1 |
| **190** | Labrador | 140 | 0 | 0 | 30,5 kg | L2-4 |  | 1 |
| **191** | Maltese | 108 | 0 | 1 | 6,7 kg | Th12-L1, L2/3 |  | 1 |
| **192** | Mixed | 132 | 0 | 0 | 8,5 kg | L4-L6 |  | 1 |
| **193** | Mixed | 91 | 1 | 1 |  | Th11-13 |  | 1 |
| **194** | Mixed | 91 | 0 | 1 | 9,0 kg | L2/L3 |  | 1 |
| **195** | Mixed | 63 | 1 | 1 | 6,6 kg | Th12/13 |  | 1 |
| **196** | Mixed | 41 | 1 | 0 | 5,5 kg | Th12-L1 |  | 1 |
| **197** | Mixed | 67 | 1 | 0 | 6,4 kg | L2/3, L3/4 |  | 1 |
| **198** | Mixed | 149 | 1 | 1 | 24,0 kg | L2/L3 |  | 1 |
| **199** | Mixed | 99 | 1 | 1 | 8,7 kg | L4/5 |  | 1 |
| **200** | Mixed | 84 | 1 | 1 | 24,0 kg | L1/L2 |  | 1 |
| **201** | Mixed | 166 | 0 | 1 | 46,9 kg | Th13/L1 |  | 1 |
| **202** | Mixed | 63 | 0 | 1 | 8,0 kg | L2-L5 |  | 1 |
| **203** | Mixed | 84 | 0 | 1 | 9,5 kg | Th13/L1, L2/3 |  | 1 |
| **204** | Mixed | 87 | 1 | 1 | 5,1 kg | Th11-12 |  | 1 |
| **205** | Mixed | 74 | 0 | 1 | 7,0 kg | L1/L2 |  | 1 |
| **206** | Mixed | 76 | 0 | 0 | 5,8 kg | Th12/13 |  | 1 |
| **207** | Mixed | 96 | 0 | 1 | 20,0 kg | L2/L3 |  | 1 |
| **208** | Mixed | 105 | 0 | 1 | 30,0 kg | L1/L2 |  | 1 |
| **209** | Mixed | 72 | 0 | 0 | 3,6 kg | Th10-L2 |  | 1 |
| **210** | Mixed | 134 | 0 | 1 | 15,0 kg | Th13-L2 |  | 1 |
| **211** | Mixed | 31 | 1 | 0 | 16,5 kg | L1/2 |  | 1 |
| **212** | Mixed | 58 | 0 | 0 | 18,5 kg | Th10-Th13 |  | 1 |
| **213** | Mixed | 88 | 1 | 1 | 8,3 kg | Th13/L1 |  | 1 |
| **214** | Mixed | 94 | 0 | 1 | 28,6 kg | L6/7 |  | 1 |
| **215** | Mixed | 42 | 0 | 0 | 19,8 kg | Th11/12 |  | 1 |
| **216** | Mixed | 49 | 0 | 1 | 30,9 kg | Th12-L2 |  | 1 |
| **217** | Mixed | 69 | 0 | 1 | 13,0 kg | Th11-Th13 |  | 1 |
| **218** | Mixed | 38 | 0 | 1 | 22,0 kg | Th12/13 |  | 1 |
| **219** | Mixed | 32 | 0 | 0 | 23,2 kg | Th13-L3 |  | 1 |
| **220** | Mixed | 61 | 1 | 0 | 10,0 kg | Th13-L2 |  | 1 |
| **221** | Mixed | 131 | 1 | 1 | 6,2 kg | Th12/13 |  | 1 |
| **222** | Mixed | 103 | 1 | 0 | 13,0 kg | L1/L2 |  | 1 |
| **223** | Old English Bulldog | 63 | 0 | 0 |  | Th13/L1 |  | 1 |
| **224** | Patterdale Terrier | 73 | 1 | 0 | 8,4 kg | Th11/12 |  | 1 |
| **225** | Pekinese | 52 | 0 | 1 | 7,5 kg | L5/L6 |  | 1 |
| **226** | Pekinese | 33 | 0 | 0 | 6,7 kg | L2-L4 |  | 1 |
| **227** | Poodle | 124 | 1 | 1 | 5,0 kg | Th12/Th13 |  | 1 |
| **228** | Pug | 43 | 0 | 0 | 18,5 kg | L1-L4 |  | 1 |
| **229** | Pug | 70 | 1 | 0 | 7,8 kg | Th12-L1 |  | 1 |
| **230** | Shi Tzu | 95 | 0 | 1 | 7,4 kg | L1/L2 |  | 1 |
| **231** | Shi Tzu | 35 | 1 | 0 | 6,3 kg | L2/L3 |  | 1 |
| **232** | Terrier | 105 | 0 | 1 | 8,2 kg | Th12-L1 |  | 1 |
| **233** | Weimaraner | 98 | 0 | 1 | 39,7 kg | L2/L3 |  | 1 |
| **234** | Yorkshire Terrier | 40 | 0 | 0 | 2,6 kg | Th12/13 |  | 1 |
| **235** | Yorkshire Terrier | 36 | 0 | 0 | 3,8 kg | L1/2 |  | 1 |
| **236** |  | 76 | 0 | 1 | 40,0 kg | L3/L4 |  | 1 |
| **237** |  | 60 | 0 | 0 | 24,0 kg | L2/L3 |  | 1 |
| **238** |  | 12 | 1 | 0 |  | L3/L4 |  | 1 |
| **239** |  | 108 | 0 | 1 |  | L1/L2 |  | 1 |
| **240** |  | 57 | 0 | 0 |  | Th12/13 |  | 1 |
| **241** |  | 144 | 1 | 1 |  | Th12/13 |  | 1 |
| **242** |  | 114 | 1 | 0 |  | L3/L4 |  | 1 |
| **243** |  | 97 | 0 | 0 |  | L2/L3 |  | 1 |
| **244** |  | 45 | 0 | 0 |  | Th10-L1 |  | 1 |
| **245** |  | 59 | 1 | 0 |  | L1/L2 |  | 1 |
| **246** |  | 84 | 1 | 1 |  | Th13/L1 |  | 1 |
| **247** |  | 45 | 0 | 1 |  | Th11/12 |  | 1 |
| **248** |  | 74 | 0 | 1 |  | L2/L3 |  | 1 |
| **249** |  | 84 | 1 | 1 |  | L1/L2 |  | 1 |
| **250** |  | 105 | 0 | 0 |  | L2/L3 |  | 1 |
| **251** |  | 72 | 1 | 1 |  | L1/L2 |  | 1 |
| **252** |  | 44 | 0 | 0 |  | L4/L5 |  | 1 |
| **253** |  | 101 | 0 | 0 |  | Th11/Th12 |  | 1 |
| **254** |  | 86 | 1 | 0 |  | Th12/13 |  | 1 |
| **255** |  | 108 | 0 | 0 |  | Th12/13 |  | 1 |
| **256** |  | 80 | 0 | 0 |  | L3/L4 |  | 1 |
| **257** |  | 144 | 1 | 1 |  | L1/L2 |  | 1 |
| **258** |  | 36 | 1 | 1 |  | Th12/13 |  | 1 |
| **259** |  | 97 | 1 | 1 |  | L4/L5 |  | 1 |
| **260** |  | 120 | 0 | 0 |  | L2/L3 |  | 1 |
| **261** |  | 96 | 1 | 1 |  | Th13/L1 |  | 1 |
| **262** |  | 84 | 0 | 0 |  | L1/L2 |  | 1 |
| **263** |  | 70 | 1 | 1 |  | Th12/13 |  | 1 |
| **264** |  | 54 | 0 | 0 |  | Th13/L1 |  | 1 |
| **265** |  | 125 | 1 | 1 |  | Th13/L1 |  | 1 |
| **266** |  | 60 | 0 | 1 |  | L1/L2 |  | 1 |
| **267** |  | 79 | 0 | 0 |  | Th13/L1 |  | 1 |
| **268** |  | 36 | 1 | 1 |  | Th13/L1 |  | 1 |
| **269** |  | 87 | 0 | 1 |  | Th13/L1 |  | 1 |
| **270** |  | 61 | 0 | 0 |  | L2/L3 |  | 1 |
| **271** |  | 50 | 0 | 0 |  | L3/L4 |  | 1 |
| **272** |  | 55 | 1 | 0 |  | Th13/L1 |  | 1 |
| **273** |  | 116 | 1 | 1 |  | Th12/13 |  | 1 |
| **274** |  | 91 | 1 | 1 |  | L4/L5 |  | 1 |
| **275** |  | 44 | 0 | 1 |  | L1/L2 |  | 1 |
| **276** |  | 56 | 0 | 1 |  | Th11/12 |  | 1 |
| **277** |  | 82 | 0 | 1 |  | L2/L3 |  | 1 |
| **278** |  | 144 | 0 | 1 |  | Th13/L1 |  | 1 |
| **279** |  | 123 | 1 | 0 |  | L3/L4 |  | 1 |
| **280** |  | 110 | 1 | 1 |  | Th3/4 |  | 1 |
| **281** |  | 84 | 1 | 0 |  | Th12/13 |  | 1 |
| **282** |  | 60 | 1 | 1 |  | Th13/L1 |  | 1 |
| **283** |  | 60 | 0 | 1 |  | Th12/13 |  | 1 |
| **284** |  | 96 | 1 | 1 |  | Th13/L1 |  | 1 |
| **285** |  | 96 | 0 | 1 |  | Th13/L1 |  | 2 |
| **286** |  | 60 | 0 | 0 |  | L2/L3 |  | 2 |
| **287** |  | 146 | 0 | 0 |  | L2/L3 |  | 2 |
| **288** |  | 91 | 0 | 1 | 36,0 kg | Th13/L1 |  | 2 |
| **289** |  | 144 | 0 | 0 |  | L1/L2 |  | 2 |
| **290** |  | 96 | 0 | 1 |  | Th13/L1 |  | 2 |
| **291** |  | 48 | 0 | 1 |  | L1/L2 |  | 2 |
| **292** |  | 73 | 0 | 1 |  | L1/L2 |  | 2 |
| **293** |  | 116 | 0 | 0 |  | L2/L3 |  | 2 |
| **294** |  | 132 | 0 | 1 |  | Th12/13 |  | 2 |
| **295** |  | 84 | 1 | 1 |  | L2/L3 |  | 2 |
| **296** |  | 108 | 0 | 0 | 35,0 kg | Th13/L1 |  | 2 |
| **297** |  | 108 | 0 | 1 |  | Th12/13 |  | 2 |
| **298** |  | 144 | 1 | 1 |  | Th9/10 |  | 2 |
| **299** |  | 94 | 0 | 1 |  | Th13/L1 |  | 2 |
| **300** |  | 111 | 0 | 0 |  | L1/L2 |  | 2 |
| **301** |  | 130 | 0 | 1 |  | Th13/L1 |  | 2 |
| **302** |  | 137 | 1 | 1 |  | Th13/L1 |  | 2 |
| **303** |  | 134 | 0 | 0 | 36,0 kg | Th13/L1 |  | 2 |
| **304** |  | 98 | 0 | 0 |  | Th13/L1 |  | 2 |
| **305** |  | 139 | 0 | 0 |  | Th12/13 |  | 2 |
| **306** |  | 79 | 0 | 1 |  | Th11/12 |  | 2 |
| **307** |  | 104 | 0 | 0 |  | Th13/L1 |  | 2 |
| **308** |  | 115 | 0 | 1 |  | L1/L2 |  | 2 |
| **309** |  | 100 | 1 | 1 |  | L1/L2 |  | 2 |
| **310** |  | 144 | 0 | 1 |  | Th12/13 |  | 2 |
| **311** |  | 145 | 1 | 1 |  | Th12/13 |  | 2 |
| **312** |  | 60 | 1 | 0 |  | Th13/L1 |  | 2 |
| **313** |  | 146 | 0 | 1 |  | Th13/L1 |  | 2 |
| **314** |  | 123 | 0 | 0 |  | L1/L2 |  | 2 |
| **315** |  | 71 | 0 | 0 |  | Th13/L1 |  | 2 |
| **316** |  | 89 | 0 | 0 |  | Th12/13 |  | 2 |
| **317** |  | 94 | 0 | 0 |  | Th13/L1 |  | 2 |
| **318** |  | 67 | 0 | 0 |  | Th12/13 |  | 2 |
| **319** |  | 115 | 1 | 1 |  | Th13/L1 |  | 2 |
| **320** |  | 99 | 0 | 0 |  | Th12/13 |  | 2 |
| **321** |  | 90 | 0 | 0 | 20,0 kg | Th12/13 |  | 2 |
| **322** |  | 97 | 0 | 1 |  | Th13/L1 |  | 2 |
| **323** | Beagle | 0 | 1 | 0 | 19,0 kg | T3-L3 | Nephroblastoma | 3 |
| **324** | Boxer | 41 | 0 | 1 | 25,0 kg | Th11-13 | Lymphoma | 3 |
| **325** | Boxer | 84 | 1 | 0 | 27,0 kg | Th3/4 | Meningioma | 3 |
| **326** | Bullmastiff | 132 | 1 | 0 | 40,0 kg | Th12 | Fibrosarcoma | 3 |
| **327** | Chihuahua | 36 | 1 | 0 | 3,0 kg | Th7-9, Th10-L1, L4-L5 | Round Cell Tumor | 3 |
| **328** | German Shepherd | 141 | 0 | 1 | 44,0 kg | Th12 | Hemangiosarcoma | 3 |
| **329** | German Shepherd | 77 | 1 | 0 | 22,0 kg | Th7 | Hemangioma | 3 |
| **330** | Greyhound | 110 | 0 | 1 | 24,0 kg | L6-7 | Metastases from prostate carcinoma | 3 |
| **331** | Jack Russell Terrier | 40 | 1 | 1 | 6,5 kg | L1-4 | Osteosarcoma | 3 |
| **332** | Labrador Retriever | 72 | 0 | 1 | 31,0 kg | L4-S3 | Lymphoma | 3 |
| **333** | Labrador Retriever | 56 | 1 | 0 | 27,0 kg | T5 | Hemangioma | 3 |
| **334** | Labrador Retriever | 131 | 0 | 0 | 36,0 kg | multifocal | Lymphoma | 3 |
| **335** | Mixed | 151 | 1 | 1 |  | L2-3 | Metastases of a mammary tumor | 3 |
| **336** | Mixed | 62 | 0 | 1 | 29,5 kg | Th12/13 | Meningioma | 3 |
| **337** | Mixed | 126 | 1 | 1 | 28,3 kg | Th12 | Hemangiosarcoma | 3 |
| **338** | Samoyed | 102 | 1 | 0 | 30,0 kg | L4-S3 | Metastases of a mammary tumor | 3 |
| **339** | Springer Spaniel | 120 | 0 | 1 | 24,0 kg | T3-L3 | Osteosarcoma | 3 |
| **340** | Staffordshire Bull Terrier | 77 | 0 | 1 | 25,0 kg | T3-L3 | Multiple Myeloma | 3 |
| **341** | Cavalier Kings Charles Spaniel | 112 | 1 | 0 | 7,0 kg | thoracolumbar |  | 4 |
| **342** | Cavalier Kings Charles Spaniel |  | 0 | 0 | 11,0 kg | thoracolumbar |  | 4 |
| **343** | Cavalier Kings Charles Spaniel | 60 | 1 | 0 | 10,0 kg | thoracolumbar |  | 4 |
| **344** | Cavalier Kings Charles Spaniel | 60 | 0 | 0 | 13,0 kg | thoracolumbar |  | 4 |
| **345** | Cavalier Kings Charles Spaniel | 40 | 1 | 0 | 8,0 kg | thoracolumbar |  | 4 |
| **346** | Cavalier Kings Charles Spaniel | 86 | 0 | 0 | 11,0 kg | thoracolumbar |  | 4 |
| **347** | Chihuahua | 117 | 1 | 0 | 3,5 kg | thoracolumbar |  | 4 |
| **348** | Chihuahua | 91 | 0 | 0 | 2,24 kg | thoracolumbar |  | 4 |
| **349** | Chihuahua | 108 | 0 | 0 | 3,9 kg | thoracolumbar |  | 4 |
| **350** | French Bulldog | 141 | 0 | 0 | 14,6 kg | thoracolumbar |  | 4 |
| **351** | Jack Russel Terrier | 118 | 0 | 0 | 10,7 kg | thoracolumbar |  | 4 |
| **352** | Mixed | 69 | 0 | 0 | 5,6 kg | thoracolumbar |  | 4 |
| **353** | Pug | 54 | 0 | 1 | 9,3 kg | thoracolumbar |  | 4 |
| **354** | Alaskan Malamute | 116 | 0 | 1 | 41,1 kg | Th13 /L1 |  | 5 |
| **355** | American Bulldog | 72 | 1 | 0 | 30,3 kg | Th13 /L1 |  | 5 |
| **356** | Appenzell Mountain Dog | 99 | 0 | 0 | 29,3 kg | Th13 /L1 |  | 5 |
| **357** | Appenzell Mountain Dog | 86 | 0 | 0 | 30,8 kg | Th12/13 |  | 5 |
| **358** | Australian Shepherd | 111 | 1 | 1 | 18,4 kg | Th12/13 |  | 5 |
| **359** | Australian Shepherd | 99 | 1 | 1 | 18,4 kg | L1/L2 |  | 5 |
| **360** | Belgian Shepherd | 33 | 0 | 0 | 28,0 kg | Th11-L2 |  | 5 |
| **361** | Bernese Mountain Dog | 59 | 1 | 1 | 35,2 kg | L1/2-L5 |  | 5 |
| **362** | Bichon Frise | 83 | 0 | 1 | 8,0 kg | Th11-13 |  | 5 |
| **363** | Bichon Frise | 44 | 1 | 0 | 5,0 kg | L2 |  | 5 |
| **364** | Border Collie | 73 | 1 | 0 | 20,5 kg | Th 12/13 |  | 5 |
| **365** | Border Collie | 72 | 1 | 1 | 21,8 kg | Th 12/13 |  | 5 |
| **366** | Border Collie | 123 | 1 | 0 | 19,5 kg | Th12/13 |  | 5 |
| **367** | Border Collie | 132 | 1 | 1 | 20,0 kg | Th13/L1 |  | 5 |
| **368** | Border Collie | 72 | 0 | 0 | 23,0 kg | Th13/L1 |  | 5 |
| **369** | Border Collie | 81 | 0 | 1 | 23,0 kg | Th12/13 |  | 5 |
| **370** | Border Collie | 114 | 0 | 0 | 21,0 kg | Th12/13 |  | 5 |
| **371** | Border Collie | 80 | 1 | 0 | 20,0 kg | Th12/13 |  | 5 |
| **372** | Border Terrier | 51 | 1 | 1 | 7,0 kg | Th12/13 |  | 5 |
| **373** | Boxer | 57 | 1 | 1 | 32,0 kg | Th13/L1 |  | 5 |
| **374** | Boxer | 96 | 1 | 1 | 34,0 kg | L3/4 |  | 5 |
| **375** | Boxer | 76 | 1 | 1 | 31,8 kg | Th10/11 |  | 5 |
| **376** | Boxer | 48 | 1 | 0 | 23,5 kg | L1-3 |  | 5 |
| **377** | Boxer | 63 | 0 | 0 | 33,0 kg | Th13 /L1 |  | 5 |
| **378** | Boxer | 40 | 1 | 1 | 42,0 kg | Th12-L1 |  | 5 |
| **379** | Briard | 63 | 1 | 0 | 29,5 kg | Th13 /L1 |  | 5 |
| **380** | Bulldog | 18 | 0 | 0 | 26,0 kg | Th7-L1 |  | 5 |
| **381** | Collie | 72 | 1 | 0 | 15,0 kg | Th13/L1 |  | 5 |
| **382** | Dalmatian | 68 | 0 | 0 | 36,0 kg | L3 |  | 5 |
| **383** | Dobermann | 139 | 0 | 1 | 38,0 kg | Th12/13 |  | 5 |
| **384** | German Shepherd | 75 | 1 | 1 | 32,0 kg | L2/3 |  | 5 |
| **385** | English Bulldog | 75 | 1 | 1 | 19,5 kg | L2-L5 |  | 5 |
| **386** | Golden Retriever | 60 | 1 | 1 | 30,0 kg | Th11-13 |  | 5 |
| **387** | Golden Retriever | 66 | 1 | 0 | 30,0 kg | Th13/L1 |  | 5 |
| **388** | Golden Retriever | 28 | 1 | 0 | 28,0 kg | L1/2 |  | 5 |
| **389** | Great Dane | 57 | 1 | 1 | 42,0 kg | L3-L4 |  | 5 |
| **390** | Irish Wolfhound | 42 | 0 | 1 | 72,0 kg | L4-5 |  | 5 |
| **391** | Jack Russell Terrier | 129 | 0 | 1 | 8,4 kg | L1/2 |  | 5 |
| **392** | Jack Russell Terrier | 78 | 0 | 1 | 7,0 kg | Th13/L1 |  | 5 |
| **393** | Labradoodle | 124 | 1 | 1 | 25,8 kg | Th12/13 |  | 5 |
| **394** | Labrador Retriever | 27 | 1 | 1 | 30,0 kg | Th 12/13 |  | 5 |
| **395** | Labrador Retriever | 8 | 1 | 0 | 35,1 kg | Th9/10 |  | 5 |
| **396** | Labrador Retriever | 52 | 1 | 1 | 20,0 kg | Th11/Th12 |  | 5 |
| **397** | Labrador Retriever | 48 | 0 | 1 | 39,0 kg | Th13 |  | 5 |
| **398** | Labrador Retriever | 120 | 0 | 1 | 33,0 kg | L1-L6 |  | 5 |
| **399** | Labrador Retriever | 51 | 0 | 1 | 30,0 kg | Th12/13 |  | 5 |
| **400** | Labrador Retriever | 76 | 1 | 1 | 27,0 kg | Th12/13 |  | 5 |
| **401** | Labrador Retriever | 72 | 0 | 1 | 34,0 kg | Th12/13 |  | 5 |
| **402** | Labrador Retriever | 30 | 0 | 1 | 28,0 kg | Th13 |  | 5 |
| **403** | Labrador Retriever | 52 | 0 | 1 | 37,0 kg | Th12/13 |  | 5 |
| **404** | Labrador Retriever | 132 | 1 | 0 | 31,0 kg | Th12/13 |  | 5 |
| **405** | Labrador Retriever | 80 | 1 | 1 | 28,0 kg | Th13/L1 |  | 5 |
| **406** | Labrador Retriever | 66 | 0 | 0 | 40,0 kg | L2/L3 |  | 5 |
| **407** | Labrador Retriever | 50 | 1 | 0 | 35,0 kg | Th13 /L1 |  | 5 |
| **408** | Labrador Retriever | 84 | 1 | 1 | 36,5 kg | Th11/12 |  | 5 |
| **409** | Labrador Retriever | 20 | 1 | 0 |  | L2/3 |  | 5 |
| **410** | Lhaso Apso | 48 | 0 | 0 | 8,0 kg | Th11/12 |  | 5 |
| **411** | Lurcher | 120 | 1 | 1 | 15,0 kg | Th12/13 |  | 5 |
| **412** | Lurcher | 84 | 1 | 1 | 20,0 kg | L2/L3 |  | 5 |
| **413** | Malinois | 86 | 1 | 0 | 26,0 kg | Th12/13 |  | 5 |
| **414** | Mastiff | 27 | 1 | 0 | 60,6 kg | Th12/13 |  | 5 |
| **415** | Mixed | 52 | 1 | 0 | 19,8 kg | Th 12/13 |  | 5 |
| **416** | Mixed | 105 | 0 | 1 | 17,0 kg | Th11/12 |  | 5 |
| **417** | Mixed | 79 | 0 | 0 | 38,0 kg | Th13 /L1 |  | 5 |
| **418** | Mixed | 104 | 1 | 1 |  | Th12/13 |  | 5 |
| **419** | Mixed | 83 | 1 | 1 |  | L1/L2 |  | 5 |
| **420** | Mixed | 160 | 1 | 1 | 25,4 kg | L2-L4 |  | 5 |
| **421** | Mixed | 73 | 0 | 1 | 11,0 kg | Th12/13 |  | 5 |
| **422** | Mixed | 90 | 0 | 1 | 25,0 kg | Th13 /L1 |  | 5 |
| **423** | Mixed | 102 | 0 | 0 | 33,0 kg | L2/3 |  | 5 |
| **424** | Mixed | 86 | 0 | 1 | 16,0 kg | Th10/11 |  | 5 |
| **425** | Mixed | 120 | 0 | 1 | 15,0 kg | Th11/12 |  | 5 |
| **426** | Mixed | 72 | 0 | 1 | 24,0 kg | Th12/13 |  | 5 |
| **427** | Mixed | 72 | 1 | 1 | 24,0 kg | L2 |  | 5 |
| **428** | Mixed | 72 | 1 | 1 | 21,0 kg | Th11-13 |  | 5 |
| **429** | Mixed | 108 | 0 | 0 | 20,0 kg | Th13/L1 |  | 5 |
| **430** | Mixed | 48 | 1 | 0 | 21,0 kg | Th10/11 |  | 5 |
| **431** | Mixed | 75 | 0 | 0 | 28,0 kg | Th10/11 |  | 5 |
| **432** | Mixed | 142 | 0 | 1 | 26,0 kg | Th9/10 |  | 5 |
| **433** | Pug | 55 | 1 | 1 | 10,4 kg | L3/4 |  | 5 |
| **434** | Prager Rattler | 80 | 1 | 0 | 4,6 kg | Th12/13 |  | 5 |
| **435** | Poodle | 101 | 0 | 1 |  | Th13 /L1 |  | 5 |
| **436** | Dachshund | 74 | 0 | 0 | 7,2 kg | Th13 /L1 |  | 5 |
| **437** | Rhodesian Ridgeback | 19 | 1 | 0 |  | Th1/2 |  | 5 |
| **438** | Rottweiler | 57 | 0 | 0 | 40,0 kg | Th2-6 |  | 5 |
| **439** | Staffordshire Bull Terrier | 76 | 0 | 1 | 30,0 kg | Th10/11 |  | 5 |
| **440** | Staffordshire Bull Terrier | 90 | 0 | 0 | 30,0 kg | Th11 |  | 5 |
| **441** | Staffordshire Bull Terrier | 48 | 0 | 1 | 30,0 kg | Th11-13 |  | 5 |
| **442** | Staffordshire Bull Terrier | 93 | 0 | 1 | 25,0 kg | L2-4 |  | 5 |
| **443** | Staffordshire Bull Terrier | 64 | 0 | 1 | 19,0 kg | Th12/13, L1-L3 |  | 5 |
| **444** | Staffordshire Bull Terrier | 72 | 0 | 1 | 20,0 kg | L2 |  | 5 |
| **445** | Staffordshire Bull Terrier | 19 | 1 | 1 | 23,0 kg | Th12/13 |  | 5 |
| **446** | Staffordshire Bull Terrier | 105 | 0 | 1 | 25,0 kg | Th13/L1 |  | 5 |
| **447** | Staffordshire Bull Terrier | 96 | 0 | 1 | 23,0 kg | Th13/L1 |  | 5 |
| **448** | Staffordshire Bull Terrier | 48 | 1 | 0 | 16,0 kg | Th12/13 |  | 5 |
| **449** | Schnauzer | 68 | 0 | 0 | 20,0 kg | Th11-L1 |  | 5 |
| **450** | Shi Tzu | 8 | 0 | 1 | 6,0 kg | Th3/4 |  | 5 |
| **451** | Shi Tzu | 60 | 0 | 0 | 7,0 kg | Th7/8 |  | 5 |
| **452** | Springer Spaniel | 84 | 0 | 1 | 21,0 kg | L1/2 |  | 5 |
| **453** | Terrier | 106 | 0 | 1 | 15,4 kg | Th12/13 |  | 5 |
| **454** | Tibet Terrier | 60 | 1 | 1 | 10,3 kg | Th13 /L1 |  | 5 |
| **455** | Tibet Terrier | 123 | 0 | 1 | 14,5 kg | Th12 |  | 5 |
| **456** | Toy Poodle | 99 | 0 | 1 | 5,0 kg | L2/3 |  | 5 |
| **457** | Whippet | 64 | 0 | 0 | 13,0 kg | Th13 /L1 |  | 5 |
| **458** | Whippet | 109 | 1 | 0 | 20,0 kg | Th12/13 |  | 5 |
| **459** | Whippet | 57 | 1 | 1 | 12,0 kg | Th12/13 |  | 5 |
| **460** | Miniature Pinscher | 83 | 0 | 0 |  | Th13 /L1 |  | 5 |
| **461** | Pomeranian | 93 | 1 | 0 | 5,1 kg | Th 11/12 |  | 5 |
| **462** | American Pittbul | 38 | 1 | 0 | 28,3 kg |  |  | healthy |
| **463** | Australian Shepherd | 122 | 1 | 0 | 16,0 kg |  |  | healthy |
| **464** | Bearded Collie | 126 | 0 | 0 | 23,3 kg |  |  | healthy |
| **465** | Cocker Spaniel | 62 | 1 | 0 | 13,1 kg |  |  | healthy |
| **466** | Golden Retriever | 131 | 0 | 1 | 38,7 kg |  |  | healthy |
| **467** | Husky | 60 | 0 | 0 | 23,5 kg |  |  | healthy |
| **468** | Lurcher | 107 | 0 | 1 | 30,0 kg |  |  | healthy |
| **469** | Malinois | 37 | 1 | 1 | 21,4 kg |  |  | healthy |
| **470** | Mixed | 138 | 1 | 1 | 12,7 kg |  |  | healthy |
| **471** | Mixed | 94 | 0 | 0 | 21,9 kg |  |  | healthy |
| **472** | Mixed | 101 | 0 | 1 | 42,4 kg |  |  | healthy |
| **473** | Mixed | 15 | 0 | 0 | 31,4 kg |  |  | healthy |
| **474** | Mixed | 118 | 0 | 1 | 13,0 kg |  |  | healthy |
| **475** | Mixed | 51 | 1 | 1 | 6,0 kg |  |  | healthy |
| **476** | Rhodesian Ridgeback | 87 | 0 | 0 | 41,7 kg |  |  | healthy |
| **477** |  | 132 | 1 | 1 | 15,0 kg |  |  | healthy |
| **478** |  | 80 | 1 | 1 | 10,0 kg |  |  | healthy |
| **479** |  | 24 | 0 | 1 | 29,0 kg |  |  | healthy |
| **480** |  | 99 | 0 | 1 | 27,0 kg |  |  | healthy |
| **481** |  | 29 | 0 | 0 | 21,0 kg |  |  | healthy |
| **482** |  | 116 | 0 | 1 | 12,0 kg |  |  | healthy |
| **483** |  | 37 | 0 | 0 | 13,0 kg |  |  | healthy |
| **484** |  | 31 | 0 | 1 | 3,0 kg |  |  | healthy |
| **485** |  | 61 | 0 | 1 | 9,0 kg |  |  | healthy |
| **486** |  | 110 | 0 | 1 | 27,0 kg |  |  | healthy |
| **487** |  | 3 | 1 | 0 | 15,0 kg |  |  | healthy |
| **488** |  | 32 | 1 | 0 | 15,0 kg |  |  | healthy |
| **489** |  | 72 | 1 | 1 | 26,0 kg |  |  | healthy |
| **490** |  | 4 | 1 | 0 | 4,0 kg |  |  | healthy |
| **491** |  | 126 | 0 | 1 | 32,0 kg |  |  | healthy |
| **492** |  | 71 | 0 | 1 | 24,0 kg |  |  | healthy |
| **493** |  | 32 | 0 | 0 | 10,0 kg |  |  | healthy |
| **494** |  | 126 | 0 | 1 | 14,0 kg |  |  | healthy |
| **495** |  | 65 | 0 | 1 | 11,0 kg |  |  | healthy |
| **496** |  | 13 | 1 | 0 | 20,0 kg |  |  | healthy |
| **497** |  | 20 | 1 | 1 | 8,0 kg |  |  | healthy |
| **498** |  | 2 | 1 | 0 | 1,0 kg |  |  | healthy |
| **499** |  | 124 | 0 | 1 | 12,0 kg |  |  | healthy |
| **500** |  | 39 | 0 | 0 | 10,0 kg |  |  | healthy |
