# Supplementary material for: Using Deep Learning to Detect Spinal Cord Diseases on Thoracolumbar Magnetic Resonance Images of Dogs
Source: Front Vet Sci. 2021 Nov 2;8:721167. doi: 10.3389/fvets.2021.721167 (PMC8593183; doi:10.3389/fvets.2021.721167)
Supplement: Supplementary file 2 [file Table_2.docx]

**Supplementary Table 2:** **Test results of the Convolutional Neural Network (CNN). 7695 images of 125 dogs were used and evaluated for CNN testing.**

*sag* = sagittal; *tra* = transverse; *FCE* = fibrocartilaginous embolism; *ANNPE* = acute non-compressive nucleus pulposus extrusion.

*For each label assigned by the system, a decision certainty was given. To best approximate a person's decision-making process, the respective label with the highest certainty assigned per section level, sequence, and patient was included in the calculation, under the condition of a correct diagnosis. The number of certainty values for neoplasia and syringomyelia was too small.
